# Promotion, prevention and protection: interventions at the population- and community-levels for mental, neurological and substance use disorders in low- and middle-income countries

**DOI:** 10.1186/s13033-016-0060-z

**Published:** 2016-04-11

**Authors:** Inge Petersen, Sara Evans-Lacko, Maya Semrau, Margaret M. Barry, Dan Chisholm, Petra Gronholm, Catherine O. Egbe, Graham Thornicroft

**Affiliations:** Centre for Rural Health, School of Nursing and Public Health and School of Applied Human Sciences, University of KwaZulu Natal, Durban, South Africa; Centre for Global Mental Health, Institute of Psychiatry, Psychology and Neuroscience, King’s College London, London, UK; World Health Organization Collaborating Centre for Health Promotion Research, National University of Ireland Galway, Galway, Ireland; Department of Mental Health and Substance Abuse, World Health Organization, Geneva, Switzerland; School of Applied Human Sciences, University of KwaZulu Natal, Durban, South Africa; Center for Tobacco Control Research and Education, University of California, San Francisco, USA

**Keywords:** Mental health, Community, Population-level, Low- and middle-income countries

## Abstract

**Background:**

In addition to services within the health system, interventions at the population and community levels are also important for the promotion of mental health, primary prevention of mental, neurological and substance use (MNS) disorders, identification and case detection of MNS disorders; and to a lesser degree treatment, care and rehabilitation. This study aims to identify “*best practice*” and “*good practice*” interventions that can feasibly be delivered at these population- and community-levels in low- and middle-income countries (LMICs), to aid the identification of resource efficiencies and allocation in LMICs.

**Methods:**

A narrative review was conducted given the wide range of relevant interventions. Expert consensus was used to identify “*best practice*” at the population-level on the basis of existing quasi-experimental natural experiments and cost effectiveness, with small scale emerging and promising evidence comprising “*good practice*”. At the community-level, using expert consensus, the ACE (Assessing Cost-Effectiveness in Prevention Project) grading system was used to differentiate “*best practice*” interventions with sufficient evidence from “*good practice*” interventions with limited but promising evidence.

**Results:**

At the population-level, laws and regulations to control alcohol demand and restrict access to lethal means of suicide were considered “*best practice*”. Child protection laws, improved control of neurocysticercosis and mass awareness campaigns were identified as “*good practice”*. At the community level, socio-emotional learning programmes in schools and parenting programmes during infancy were identified as “*best practice*”. The following were all identified as “*good practice”*: Integrating mental health promotion strategies into workplace occupational health and safety policies; mental health information and awareness programmes as well as detection of MNS disorders in schools; early child enrichment/preschool educational programs and parenting programs for children aged 2–14 years; gender equity and/or economic empowerment programs for vulnerable groups; training of gatekeepers to identify people with MNS disorders in the community; and training non-specialist community members at a neighbourhood level to assist with community-based support and rehabilitation of people with mental disorders.

**Conclusion:**

Interventions provided at the population- and community-levels have an important role to play in promoting mental health, preventing the onset, and protecting those with MNS disorders. The importance of inter-sectoral engagement and the need for further research on interventions at these levels in LMICs is highlighted.

**Electronic supplementary material:**

The online version of this article (doi:10.1186/s13033-016-0060-z) contains supplementary material, which is available to authorized users.

## Background

Over two thirds of people with mental, neurological and substance use (MNS) disorders do not receive the care they need worldwide [1]. This is particularly acute in low- and middle-income countries (LMICs), which is home to three-quarters of the global burden of disease attributable to mental and neurological disorders [2].

In addition to strengthening services for mental health care provided by the health care system, for example, through programmes such as the Mental Health Gap Action Programme (mhGAP) [3, 4]; interventions at the population and community levels that promote mental health; prevent the development of MNS disorders and protect people with MNS disorders are also important. At the population-level, mechanisms for the delivery of these interventions include legislation, regulations, and public information campaigns. At the community level, these interventions are best delivered at schools, workplaces, and in neighbourhoods/community groups, requiring a setting-based approach.

In pursuit of reducing the burden of mental disorders in LMICs, identifying interventions that can be effectively and feasibly delivered at these levels is helpful to decision-makers as it highlights where resources should be allocated, besides in the health care sector (e.g. to schools or non-governmental organizations in the community). It also enables potential opportunities, synergies and efficiencies to be identified across multiple sectors.

This paper provides an overview of the evidence for interventions at the population- and community-levels in LMICs along the continuum of care from interventions that promote positive mental health, and primary preventative interventions which strive to prevent the onset of MNS disorders; through to identification and case detection; as well as treatment, care and rehabilitation. The evidence presented for each level is structured around these core elements of the continuum of care. The review was informed by the chapter on population and community ‘platforms’ or levels of care in the mental health volume of Disease Control Priorities (third edition) [5] as well as the mental health promotion and prevention aspects of the World Health Organization Regional framework to scale up action on mental health in the Eastern Mediterranean Region [6].

## Methods

### Population level interventions

Population level interventions are rarely evaluated using the gold standard of randomized controlled trials. More commonly used approaches are quasi-experimental natural experiments, with before-and-after data obtained from archival analysis of official statistics or surveys, and comparisons with populations who have not been exposed to the intervention, where possible. A narrative review approach was used given the need to cover a wide range of study designs and issues which do not lend themselves to a systematic review [7]. Available disorder-specific evidence on the most effective and cost-effective interventions provided by mental health volume of Disease Control Priorities (third edition) [5] was used; supplemented by a narrative review of the best evidence where necessary. The authors of this manuscript provided expert consensus on whether interventions were classified as “*best practice*” on the basis of whether evidence from quasi-experimental natural experiments was available in LMICs as well as evidence of cost-effectiveness in LMICs. “*Good practice”* interventions were identified on the basis of whether there was limited but promising local small scale studies in LMICs, and where further research was still needed.

### Community-level interventions

Studies on interventions at the community level in LMICs were identified on the same basis as for the population-level. Many of these interventions have a prevention and promotion focus, and expert consensus by the authors of this manuscript was used to evaluate effectiveness using the ACE-Prevention framework [8]. The ACE (Assessing Cost-Effectiveness in Prevention Project) grading system provides a single framework for the evaluation of evidence on clinical, public health, and behavioral interventions. Using this system, *sufficient evidence* is when there is at least one systematic review of randomized control trials (RCTs), as well as several good quality RCTs or several high-quality pseudo-RCTs using alternate allocation or another method, or non-RCTs with comparative groups to exclude chance. *Limitedevidence* is when the effect is probably not due to chance, but bias cannot be ruled out as a possible explanation for the effect. We have classified this evidence as *promising*. *Inconclusive evidence* is when there is no evidence of systematic reviews or RCTs, although there may be a few poor quality pseudo-randomized/non-RCTs with comparative groups or cohort studies.

“*Best*-*practice*” interventions were identified on the basis of whether there was sufficient evidence of their effectiveness in LMICs using the ACE grading system, evidence of their cost-effectiveness in HICs, and evidence of their feasibility in relation to cultural acceptability and capacity for scale-up in resource-constrained settings in LMICs. “*Good practice”* interventions were identified on the basis of sufficient evidence of their effectiveness in HICs and limited but promising evidence of their effectiveness in LMICs, using the ACE framework.

## Results

Details of the specific studies reviewed can be found in Additional file 1: Table S1.

### Population level

#### Legislation and regulations for promotion and primary prevention

- Reducing demand for alcohol products
  The prevention of harmful alcohol use in adults provides benefits across diseases. It can help prevent the development of alcohol use disorder and unipolar depression; other chronic non-communicable diseases, such as cardiovascular disease, diabetes, and cirrhosis of the liver; and help prevent accidental and intentional injuries or death [9].
  Evidence from HICs and LMICs indicates that the most cost-effective strategy for reducing alcohol consumption is increased taxation and/or pricing of alcohol products, followed by bans on alcohol advertising, restrictions on access to alcohol, and enforcement of drinking and driving legislation [9, 10]. Laws and regulations reducing demand for alcohol products are thus considered “*best practice*”. Raising taxes is, however, less effective in countries with lower levels of alcohol consumption where other targeted interventions, such as enforcing drunk-driving legislation and brief screening and intervention (BSI), are more effective [9]. Regulations may also be less effective in countries where alcohol can be easily acquired through the unregulated/black market/home brews.
- Restricting access to means of suicide
  Three quarters of the 804,000 deaths by suicide that are estimated globally for 2012 were from LMICs [11]. Regulations restricting access to common, regional-specific, lethal means of suicide have been effective in reducing suicide rates in HICs and LMICs [11, 12] as well as being cost-effective [13], and is thus considered “*bestpractice*”. The impact of the introduction of pesticide regulations on the reduction of suicides in Sri Lanka provides an example of how this strategy has been effectively applied in LMICs, where the suicide rate of 47 deaths per 100,000 in 1995 was halved from 1996 to 2005, with a reduction of 19 769 suicides, as a result of the banning of all WHO toxicity Class pesticides in 1995 and the banning of endosulfan, a Class II toxicity pesticide in 1998 [14]
- Other multi-sectoral public policies with promising evidence to promote mental health and prevent MNS disorders in children and adults
  During childhood, child maltreatment is a risk factor for the development of MNS disorders [15]. Sufficient evidence from HICs [16] and some limited but promising evidence from LMICs indicates that the enactment of child protection laws for children living outside of the family have health and safety benefits for these children [17] [15], although further research to assess the benefits for children within their families of origin is indicated. Limited but promising evidence from Honduras also suggests that improved control of neurocysticercosis, (a common cause of epilepsy in LMIC) through deworming of humans, vaccination of pigs, improved sanitation, meat inspection, and improved pig farming, can lead to a reduction in symptomatic epilepsy in hyperendemic populations [18]. Given that the evidence for these interventions is still limited, they are recommended as “*good practice”* at the population level.

### Information and awareness campaigns for promotion and primary prevention

Information and public awareness campaigns employ broad strategies and messages to promote mental health literacy, defined as knowledge and beliefs about mental disorders to aid their recognition, management, and prevention [19]; as well as reduce stigma and discrimination and hence help protect people with MNS disorders. They disseminate information about signs and symptoms of MNS disorders, locations where people may receive help, facts and figures about prevalence, risk factors, and evidence to combat stigmatizing beliefs.

Most information and awareness programs represent low-intensity interventions aimed at large numbers of people, often through print media, recordings, radio, television, cinema, mobile phones, and the Internet [20, 21]. There is a growing evidence base from HICs of the effectiveness of large-scale efforts for increasing knowledge and recognition of MNS disorders [22], improving attitudes [23, 24], and reducing discrimination in a cost-effective manner [25–30]. Although information and awareness programs often cover a broad range of MNS disorders, most focus on mental rather than neurological disorders, with one randomized controlled trial from Hong Kong showing that exposing individuals to information about dementia through vignettes led to a statistically significant reduction in stigma [31].

With regard to anti-stigma interventions specifically, a systematic review of interventions in HICs by Clement and colleagues showed that mass media awareness programmes may reduce prejudice; although fewer studies have investigated their effects on actual discrimination [21]. A review by Corrigan and colleagues examined anti-stigma approaches that incorporated elements of education, protest, or contact [32]. In-person contact interventions yielded the greatest effect in adults; education was most effective among adolescents. One challenge is to deliver these types of interventions on a mass scale to the public, with some evidence of the effectiveness of virtual contact via film or video [33]; as well as the feasibility of achieving positive intergroup contact via large public events [34].

There is a paucity of evidence for the effectiveness of mass awareness programmes to improve mental health literacy and reduce stigma in LMICs. One study in the Russian Federation investigated the effectiveness of an anti-stigma computer program for improving knowledge and attitudes as well as reducing social distance among university students [35]. Students were randomized to one of three groups: a computer program group, a reading group, or a control group. At 6 months follow-up, while the reading group showed some improvement in attitudes; all stigma outcomes were significant in the computer program group.

Based on sufficient evidence from HICs and emerging evidence from LMICs, mass public awareness campaigns are recommended as “*good practice”*; with more research in LMICs being particularly needed.

#### Legislation and regulations to improve identification, treatment and care of persons with mental disorders

Evidence of the impact of legislation and regulations for improved identification, treatment and care is lacking from both HICs and LMICs. It would, however, be reasonable to assume that up-to-date mental health laws and regulations that are in line with the best practice and human rights standards such as those outlined by the WHO QualityRights [36] would promote protection of persons with mental disorders, but have not been identified as such here given the lack of evidence.

### Community level

#### Workplaces

##### *Promotion and primary prevention*

Workplace settings provide an ideal setting for the provision of promotion and prevention interventions for adults. Evidence from HICs indicates that individual and organizational level interventions improve and maintain mental health in the workplace, including screening and cognitive behavioral therapy (CBT) for preclinical symptoms of depression and anxiety to prevent the onset of these disorders [37, 38]. The evidence base from LMICs is, however, sparse. Limited but promising evidence of effectiveness of primary prevention and promotion is provided by the New SOLVE training package, developed by the International Labour Organization [39]. This package focuses on integrating mental health promotion strategies, such as stress reduction and awareness of alcohol and drug misuse, into occupational health and safety policies [39]. These integrated mental health strategies in the workplace are recommended as “*good practice”*, given limited but promising evidence, with a recommendation that more robust evidence be generated from LMICs.

##### Identification and case detection

Evidence is available from HICs on the identification and case detection of MNS disorders in the workplace. An evaluation of the APPRAND program in France found that individuals on sick leave who were screened and identified as having anxiety and depressive disorders by company health physicians, and who received an awareness-raising and referral intervention, displayed higher remission and recovery rates, compared to individuals in other centres who were not screened and who did not receive the intervention [40]. Positive effects have been reported for a mental health first aid course in Australia [41] that included training in screening for mental disorders. There have also been encouraging results in the US for migraine/headache management programs that have included screening questionnaires and educational initiatives, which resulted in an increase in the number of participants seeking help from a physician, an improvement in headache symptoms, and a reduction in absenteeism amongst those affected and the resulting employer cost burden [42, 43]. There is, however, insufficient evidence from LMICs for screening of MNS disorders in workplace settings to be recommended yet, with further research in LMICs required.

##### Treatment, care, and rehabilitation

Interventions for the treatment, care, and rehabilitation of people with MNS disorders in the workplace have been shown to be effective in HICs. For people with common mental health problems, individual therapies rather than organizational interventions have been the most effective; in particular, CBT [44–46] (either face-to-face or more questionably via computer software) [47, 48]. To a lesser extent, exercise and relaxation interventions, such as aerobic or meditation sessions, have been beneficial [49]. Independent case management by third-party specialists, such as labour experts or employment advisors, has also shown a positive impact on people with common mental disorders when combined with psychological therapies, such as CBT [46]. Multimodal interventions may be more effective than single interventions [44]. With regard to severe mental disorders, there is also sufficient evidence from HICs of the benefits of supported employment, for example, individual placement and support (IPS), in helping people obtain competitive employment [50–52]. For neurological disorders, a few studies have shown positive effects (though with mixed results) of educational and physical programs that have been implemented in workplace settings in Italy and Finland to reduce headaches and neck/shoulder pain [53–55].

Overall, evidence for the treatment, care and rehabilitation of MNS disorders in the workplace from LMICs is insufficient for recommendations to be made yet, with one RCT in South Africa showing that a workplace intervention consisting of workability assessments and workplace visits was able to facilitate return to work for stroke patients [56]. Further research is recommended in LMICs on the effectiveness of training in first-level management of acute symptoms, particularly CBT, for anxiety or depression (possibly combined with independent case management), supported employment for severe mental disorder, and educational, physical and return-to-work interventions for neurological disorders.

#### Schools

##### Promotion and primary prevention

- Information and awareness
  Examples of robust evaluations of broad information and awareness interventions addressing MNS literacy in schools are more generally available in HICs [57–59]. In LMICs, one study using a randomized control design was sourced. It was performed in rural secondary schools in Pakistan led by health care professionals, and involved a short training course for teachers, having a co-constructed educational program of lectures and several participatory activities. The study assessed changes in knowledge and attitudes 4 months after the start of the program. Improvements were noted among schoolchildren, their parents, friends, and neighbours. In the control group, there were improvements only among schoolchildren and their friends [60]. For neurological disorders, only studies in HICs could be sourced. Hip Hop Stroke, is an example of an information and awareness programme for children (8–12 years) from schools in a high risk stroke neighbourhood in the United States that showed improved knowledge of stroke symptoms and behavioral intent to call 911 [61]. Given promising emerging evidence from LMICs of the positive impact of information and awareness interventions in schools, these programmes are recommended as “*good practice”*; with further research recommended.
- Social and emotional learning (SEL) interventions
  Studies from HICs and LMICs indicate that universal SEL programs that promote social and emotional competencies can improve social and emotional functioning and academic performance in exposed children, as well as reduce risk behaviour. Systematic reviews from HICs show that universal SEL interventions in primary and post-primary schools promote children’s social and emotional functioning and academic performance, including evidence of long-term benefits [62–67]. Interventions that employ a whole-school approach where SEL is supported by a school ethos and a physical and social environment that is health enabling involving staff, students, parents, school environment and local community are most effective, and have additionally been shown to reduce bullying [68], with bullying in childhood and adolescence a risk factor for the development of mental disorders [69].
  A systematic review [70], as well as other studies [71–74] also provide sufficient evidence of the beneficial effects of universal SEL programs in LMICs. Delivery of these interventions by teachers and school counsellors through integrating social and emotional learning, including life skills development into the school curriculum, is feasible as demonstrated by the Healthwise program in South Africa [73]. However, fidelity can affect the impact of SEL interventions; teacher training, support, and supervision are needed, as is attention to the school environment [75]; suggesting that integration into a whole school approach that pays attention to contextual issues would be optimal.
  Targeted/indicated interventions for high-risk children (children having had experiences that elevate their vulnerability of developing a MNS disorder/show pre-clinical symptoms of a disorder) that promote coping and resilience, including cognitive skills training have been found to help prevent the onset of anxiety, depression, and suicide in HICs [76–78]. Several RCTs of targeted interventions for vulnerable children have also been conducted in LMICs [79]. Classroom-based interventions for vulnerable children, especially those orphaned by HIV or living in areas of conflict, have shown in some studies to improve general psychological health and coping [80–83]; however, these effects are contingent on individual variables, such as age and gender, as well as contextual variables, such as conflict, displacement, and family functioning [84], and may be better suited for children with less severe risks and difficulties [79].
  Economic analyses from HICs indicate that SEL interventions in schools are cost-effective, resulting in savings from better health outcomes, as well as reduced expenditures in the criminal justice system [85, 86]. Based on sufficient evidence as well as feasibility, universal school-based SEL interventions are recommended as “*best practice*” interventions in LMICs, and targeted school-based interventions as “*good practice”*, with more research on the role of individual and contextual variables on mental health outcomes required.

##### Identification and case detection

Many people with MNS disorders have their onset during childhood and adolescence, and these early difficulties are likely to be present in the school context. Teachers have a critical role in identifying emerging problems and taking appropriate action, with RCTs from HICs providing evidence for training in indicated screening of developmental and behavioral disorders in schools. Mental health first aid for high school teachers has been tested using a cluster RCT [87]. While data from LMICs are more limited, evidence supports the feasibility and reliability of identifying and assessing MNS disorders in primary and secondary school students [88–91]. In Haiti, a 2.5 day training program for secondary school teachers focused on recognizing, responding, and referring students at risk for MNS disorders following the earthquake in 2010. The intervention was associated with improvements in knowledge, attitudes, and recognition of MNS problems [92]. In terms of neurological disorders, in Chandigarh city, India, a one-off educational intervention package improved teachers’ knowledge, attitudes and skills regarding epilepsy immediately after the intervention, and at 3-month follow-up. However, it was noted that further workshops would be required for long-term benefit [93].

Given sufficient evidence from HICs as well as emerging promising evidence from LMICs, identification and case detection in schools of children with MNS disorders are recommended as “*good practice”*; with further research in LMICs required.

##### Treatment, care, and rehabilitation

There is sufficient research evidence of the effective treatment and management of people with some types of MNS disorders in schools in HICs. A meta-analysis that examined the effectiveness of various types of school-based CBT for young people with anxiety and depression showed significant reductions in symptoms overall [94]. School-based interventions for attention-deficit/hyperactivity disorder (ADHD) have been found to be promising in younger children but less so for adolescents; these interventions lack robust long-term program effectiveness data, as well as cost effectiveness data [95]. Effective ADHD interventions for academic and behavioral outcomes involve contingency management, academic intervention, and cognitive-behavioral interventions [96]. For neurological disorders, a classroom-based headache prevention program in Germany found a small but significant reduction in reported tension-type headaches 7 months following the intervention [97].

Evidence from HICs indicates that children with emotional and behavioural disorders benefit from classroom environments that are predictable and consistent, with clear structures and rules; such settings are associated with improved classroom, peer behavior, and enhanced learning [98]. Interventions that use direct instruction, peer tutoring, and behaviorally based procedures, such as time delay prompting, trial and error, and differential reinforcement, hold promise [99].

Evidence from LMICs for treatment, care and rehabilitation for children with MNS disorders is equivocal. An RCT of a universal school-based intervention in reducing depressive symptoms was conducted in Chile. Using CBT techniques delivered by non-specialists, this intervention comprised 11 1-h weekly and two booster classroom sessions. Although it was a universal intervention, the study analysed subgroups of young people with high depression scores. It showed no clinically significant difference between the intervention and control groups, and no evidence of effect modification by severity of symptoms [100]. There have also been a few trials of the classroom-based intervention (CBI) incorporating cognitive behavioral techniques and creative expressive elements to help children with depressive, anxiety, and posttraumatic stress disorder (PTSD) symptoms in complex emergencies in LMICs [81, 84, 101, 102]. The emerging evidence on the effectiveness of treatment of PTSD and depressive symptoms is inconsistent; with CBI having more consistent preventive benefits, particularly when risks are less severe. CBI can thus not be recommended for treatment of these conditions in conflict-affected children [79]. Given the equivocal evidence from LMICs, further research generating positive outcomes for the treatment care and rehabilitation for children with MNS disorders in schools is required before recommendations for LMICs can be made.

#### Neighbourhood/community groups

##### Primary prevention and promotion

There is an array of primary prevention and promotion interventions delivered in neighbourhood settings or through community groups. These include programs on early child enrichment/preschool educational programmes, community-based parenting, and gender and/or economic empowerment interventions.

- Child enrichment and preschool educational programs
  Robust evidence from HICs demonstrates the effectiveness and cost-effectiveness of early child enrichment and preschool educational programs (which promote cognitive stimulation and social interaction) on children’s social and emotional wellbeing, cognitive skills, problem behaviors, and school readiness [65, 103, 104]; as well as evidence of the long-term effects on school attainment, social gains, and occupational status in HICs [105]. The evidence from LMICs is promising [15, 106–109] and these interventions are thus recommended as “*good practice”*.
- Parenting interventions
  The effectiveness of parenting interventions for promoting child emotional and behavioral adjustment in HICs, particularly in infants and younger children, has been demonstrated [110], as well as the cost-effectiveness of programs for the prevention of conduct disorders [85]. There is also sufficient evidence from LMICs of the effectiveness and feasibility of parenting programs to enhance mother–child interaction during infancy for these interventions to be considered “*best practice*” [15, 111–116]. Many of these interventions are delivered at health centres or utilize a home visitation program and therefore overlap with the primary health care facility platform. The effectiveness of community parenting programs in HICs for preventing internalizing and externalizing disorders in older children (pre-school and school-going), has also been demonstrated in HICs [117] with promising evidence in LMICs [113, 118–120]; and these interventions are thus recommended as “*good practice”*.
- Gender equity and/or economic empowerment interventions
  A growing body of research indicates the feasibility and benefits for vulnerable adolescents and adults of gender equity and/or economic empowerment programs in LMICs [121–127]. Microfinance (micro-credit and micro-savings) schemes for poor people in sub-Saharan Africa that incorporate gender empowerment, health and education training components are seemingly more effective in terms of mental health benefits over stand-alone programmes [128, 129]. Given promising evidence, these programs are recommended as “*good practice”*.

##### Identification and case detection

Mental health first aid training at the community level involves training members to identify when a person is developing a mental disorder, is suicidal, or is in crisis; to know how to manage the situation; and to know appropriate facilities for referral [19]. Evidence for feasible and effective identification training programs of non-mental health workers is particularly robust for police officers and community health workers in HICs and LMICs. Given that community health workers may operate out of health centres or utilize a home visitation program, these interventions may overlap with services provided by the health system. With regard to neurological disorders, research from HICs suggests that trained community health workers can facilitate early detection of dementia in resource poor communities [130]. Moreover, if screening leads to early intervention within a year of detection, it could be associated with cost savings through reduced healthcare costs in the long run [131]. Mental health first aid (MHFA) training of community members generally has also been found to increase knowledge, reduce stigma, and increase helping behaviors in HICs. While MHFA is being rolled out in a number of LMICs, evidence of effectiveness is still required [132]. With sufficient evidence from HICs, as well as emerging promising evidence from LMICs of the effectiveness of training of non-mental health workers and community members in the identification and case detection, it is recommended as “*good practice”*; with further research in LMICs recommended.

##### Treatment, care, and rehabilitation

Policy shifts to deinstitutionalization and decentralized care in many LMICs are heightening the need for community-based treatment and rehabilitation for mental disorders. There is emerging evidence of the effectiveness of collaborative community-based care interventions using a task-sharing approach in LMICs. The Community Care for People with Schizophrenia in India (COPSI) intervention compared the effectiveness of facility-based care with a collaborative community-based approach using a multicentre RCT. Findings demonstrated that the collaborative community-based approach in combination with facility-based care was most effective and was associated with improvements in disability and symptoms [133]. Observational studies in India also suggest that community-based rehabilitation and microfinance initiatives can improve symptoms and reduce disability [134–136]. More targeted interventions, such as psycho-education [137–143]; adherence support [144, 145], and self-help groups also show promise in LMICs. Most studies focus on psychosis; however, community-based interventions can be beneficial for other MNS disorders as well. In this respect, a cluster RCT demonstrated that using community counselors for the treatment of maternal depression through a home visitation programme was associated with greater recovery [146] and a home-based care support programme for carers of people with dementia showed improved mental health outcomes for the carers [147]. These interventions are recommended as good practice in LMICs, and further research on the effectiveness of interventions using non-specialists, including traditional healers, at a neighbourhood level or through community groups are recommended.

## Discussion

Interventions identified as being “*best practice*” and “*good practice*” are summarized in Table 1.

**Table 1:** Matrix of “*Best Practice”* and “*Good Practice”* interventions

Delivery platform	Promotion and primary prevention	Identification and case detection	Treatment, care and rehabilitation
Delivery subplatform			
Population wide			
Legislation and regulation	*Laws and regulations to reduce demand for alcohol use*:		
	*Laws to restrict access to means of self-harm/suicide*		
	Child protection laws		
	Improved control of neurocysticercosis		
Information/awareness	Mass public awareness campaigns		
Community			
Workplace	Integrating mental health promotion strategies such as stress reduction and awareness of alcohol and drug misuse into occupational health and safety policies		
Schools	*Universal SEL programs*	Information and awareness	
	Targeted programmes for vulnerable children	Identification and case detection in schools of children with MNS disorders	
Neighbourhood/community groups	*Parenting programs during infancy*	Training of gatekeepers, including community health workers, police, and social workers in identification of MNS disorders, including self-harm	Training non-specialist community members at a neighbourhood level to assist with community-based support and rehabilitation of people with mental disorders
	Early child enrichment/preschool educational programs		
	Parenting programs for children ages 2–14 years		
	Gender equity and/or economic empowerment programs for vulnerable groups		

Recommendations in italics = “*best practice”*; recommendations in normal font = “*good practice*”. *MNS* mental, neurological, and substance; *SEL* socio-emotional learning

Interventions at the population level have a broad reach, promoting and protecting the mental health of the entire population through legislation, regulations, and public campaigns. There is good evidence that legislation and regulations to control alcohol demand can reduce alcohol consumption in LMICs at minimal cost, and laws and regulations restricting access to lethal means of suicide that are region-specific can reduce suicide rates in LMICs. There is also promising evidence of the benefits of mass public awareness campaigns on reducing stigma and discrimination; as well as the mental health benefits of child protection laws, and laws and regulations for improved control of neurocysticercosis in reducing epilepsy specifically.

At the community level, there is evidence from LMICs that neighbourhoods are an important setting for the delivery of primary prevention and promotion interventions. In particular, there is strong evidence that parenting programs during infancy, that promote mother–child interaction, are beneficial for long term mental health. There is also promising evidence of the mental health benefits of early child enrichment/preschool educational programs, parenting programs for older children, and gender equity and/or economic empowerment programs for vulnerable groups. Neighbourhoods are also important settings for the identification of people with MNS disorders, using trained community gatekeepers.

Schools are a particularly important setting for mental health promotion and prevention interventions in children; as well as identification. In particular, there is sufficient evidence of universal SEL programs for improving mental health outcomes in children and to a lesser degree targeted programmes in LMICs. Schools can also be used for the delivery of information and awareness programmes.

Finally, the workplace presents an opportune setting for mental health promotion and prevention activities for adults, with emerging evidence in LMICs of the benefits of integrating stress reduction programmes and awareness-raising, particularly of alcohol and drug misuse.

## Conclusion

Although much attention has historically been paid to the health sector for the delivery of mental health services, greater consideration of interventions at the population- and community-levels are necessary, particularly for the delivery of mental health promotion and prevention interventions, as well as for the early identification of mental disorders, especially in children and adolescents, and to a lesser degree, care and rehabilitation. This review has identified “best practice” and “good practice” interventions at the population and community levels; providing evidence of potential opportunities and synergies for the strengthening of mental health and human capital development across multiple sectors in LMICs. Harnessing these opportunities, however, requires awareness of mental health as a public health and social development priority and political will to engage in collaborative arrangements across different sectors.

## Additional file

10.1186/s13033-016-0060-z Evidence of primary prevention and promotion interventions at the population and community level platform.
