# Supplementary material for: Promotion, prevention and protection: interventions at the population- and community-levels for mental, neurological and substance use disorders in low- and middle-income countries
Source: Int J Ment Health Syst. 2016 Apr 11;10:30. doi: 10.1186/s13033-016-0060-z (PMC4827227; doi:10.1186/s13033-016-0060-z)
Supplement: Supplementary file 1 — 10.1186/s13033-016-0060-z Evidence of primary prevention and promotion interventions at the population and community level platform. [file 13033_2016_60_MOESM1_ESM.docx]

**Table S1: Evidence of Primary Prevention and Promotion Interventions at the Population and Community Level Platform**

| **Platform** | **Key issue** | **Quality of Evidence from HICs** | **Quality of Evidence from LMICs** |
| --- | --- | --- | --- |
| **Population-wide platform** | | | |
| **Legislation and regulation** | Raised tax/price on alcohol  Restricted access to alcohol  Bans on alcohol advertising | Sufficient evidence that raised tax/price on alcohol, restricted access and bans on alcohol advertising reduce alcohol consumption ([1](#_ENREF_1), [2](#_ENREF_2)) | Sufficient evidence that raised tax/price on alcohol, bans on alcohol advertising reduce consumption of alcohol ([1](#_ENREF_1), [2](#_ENREF_2)) |
|  | Restricting access to potential lethal means of suicide | Restricting access to commonly used lethal means of suicide e.g., Firearm control legislation, restrictions on pesticides, detoxification of domestic gas has been shown to decrease rates of suicide ([3](#_ENREF_3), [4](#_ENREF_4)) | Restricting access to commonly used lethal means of suicide e.g., Firearm control legislation, restrictions on pesticides, detoxification of domestic gas has been shown to decrease rates of suicide ([3](#_ENREF_3), [4](#_ENREF_4)) |
|  | Child protection laws | Sufficient evidence from HICs ([5](#_ENREF_5)) | Promising evidence that child protection laws for children living outside the family have health and safety benefits for these children ([6](#_ENREF_6)) |
|  | Improved control of neurocysticercosis |  | Promising evidence from LMIC that improved control of neurocysticercosis can lead to a reduction in symptomatic epilepsy in hyperendemic populations ([7](#_ENREF_7)). |
| **Information and awareness** |  | Sufficient evidence that mass media interventions reduce prejudice in the immediate, short and medium term ([8-12](#_ENREF_8)) | Promising evidence from small scale local interventions([13](#_ENREF_13)) |
| **Community-level platform** | | | |
| **Workplaces** | Individual and organizational level interventions to promote mental health and primary prevention of MNS disorders | Individual and organizational level interventions improve and maintain mental health at work, including screening and cognitive behavioral therapy for pre-clinical symptoms of depression and anxiety to prevent the onset of these disorders ([14](#_ENREF_14)) | Promising evidence of effectiveness for promoting mental health is provided by the new SOLVE training package, developed by the International Labour Organization ([15](#_ENREF_15)). This package focuses on integrating mental health promotion strategies such as stress reduction and awareness of alcohol and drug misuse into occupational health and safety policies. |
|  | Training in indicated screening of MNS disorders | Sufficient evidence for the effectiveness of screening of CMD in workplace settings ([16-19](#_ENREF_16)) |  |
|  | Supported employment for people with SMD to obtain employment | Sufficient evidence for the effectiveness and possibly cost-effectiveness of supported employment in the US and several European HICs ([20-22](#_ENREF_20)) |  |
|  | Training in first-level management of acute symptoms of MNS disorders, particularly CBT for CMD | Sufficient evidence from HICs for the effectiveness of CBT in the workplace for people with CMD ([23-25](#_ENREF_23)) |  |
| **School** | School-based mental health awareness programmes. | Sufficient evidence of information and awareness programmes that address knowledge and attitudes about mental illness ([26-28](#_ENREF_26)) | Promising evidence, including one RCT from a LMIC that was performed in rural secondary schools in Pakistan ([29](#_ENREF_29)) |
|  | Universal social and emotional learning (SEL) programmes | Sufficient evidence from HIC of the effectiveness of SEL and whole school approaches to mental health promotion in terms of children’s improved social and emotional functioning, academic performance and social wellbeing ([30-35](#_ENREF_30)) | Sufficient evidence of effectiveness of SEL programmes in schools from LMIC([36-39](#_ENREF_36)) |
|  | Targeted/indicated interventions for high-risk children (children having had experiences that elevate their vulnerability to developing a MNS disorder/ show pre-clinical symptoms of the a disorder) | Sufficient evidence that targeted programmes that promote coping skills, resilience, and cognitive skills training help to prevent the onset of anxiety, depression, and suicide in HIC ([40-42](#_ENREF_40)) | Several RCTs of targeted interventions for vulnerable children ([43](#_ENREF_43)). However, effects are contingent on individual variables, such as age and gender, as well as contextual variables, such as conflict, displacement, and family functioning ([44](#_ENREF_44)) and may be better suited for children with less severe risks and difficulties ([43](#_ENREF_43)) |
|  | Interventions supporting teachers to recognize MNS disorders | Sufficient evidence including cluster randomized trials of programmes ([45](#_ENREF_45)). | Promising evidence supporting the feasibility and reliability of identifying and assessing MNS disorders among primary and secondary school students ([46-48](#_ENREF_46)) |
|  | Treatment or management of MNS disorders in schools | Sufficient evidence for the effective treatment and / or management of MNS disorders, particularly for anxiety and depression, but also for ADHD ([49-51](#_ENREF_49)) | The evidence is inconsistent and equivocal ([44](#_ENREF_44), [52-55](#_ENREF_52)) |
| **Neighbourhood/Community groups** | Universal and targeted parenting programmes for infants | Sufficient evidence that early (0-3 years) universal and targeted parenting programmes impact positively on child emotional and behavioural adjustment ([56](#_ENREF_56)) | Sufficient evidence of the effectiveness and feasibility of programmes designed to enhance mother-child interaction during infancy ([57-62](#_ENREF_57)) |
|  | Parenting programmes for pre- and school aged children | Sufficient evidence that parenting programmes can ameliorate/prevent internalizing and externalizing problems in 0-7 year olds ([63](#_ENREF_63)) | Promising evidence of effectiveness of parenting interventions for preventing externalizing disorders and reducing risk behaviours in pre-school and school-going children (2-14 years)  ([59](#_ENREF_59), [64-66](#_ENREF_64)) |
|  | Out- of school gender equity and/or economic empowerment programmes for adolescents/young adults |  | Promising evidence of effectiveness ([67-71](#_ENREF_67)) |
|  | Child enrichment/preschool educational programmes | Sufficient evidence from HIC on beneficial impact on children’s mental health, academic and social functioning ([33](#_ENREF_33), [72-74](#_ENREF_72)) | Promising evidence of the beneficial effects of child enrichment/ preschool parenting interventions ([75-78](#_ENREF_75)) |
|  | Training non-mental health workers in identifying persons with MNS disorders | Training police, community members, spiritual leaders, social workers, and community health workers in identification and case detection can lead to improved access to care and reduced stigma ([79-84](#_ENREF_79)) | Promising evidence for effectiveness of training non mental health workers in case identification of MNS disorders in LMICs, particularly community health workers ([85](#_ENREF_85)) |
|  | Community-based treatment and care | Assertive community treatments have been found to decrease use of inpatient psychiatric services and improve symptoms and subjective quality of life and is associated with better service contact, housing and employment stability ([86-88](#_ENREF_86)) | Promising evidence that community based treatment and rehabilitation improves outcomes in people with mental illness living in LMIC ([89-97](#_ENREF_89)) |

**References**

1. Rehm J, Chisholm D, Room R, Lopez A. Alcohol. In: Jamison D, Breman J, Measham A, Alleyne G, Evans D, Jha P, et al., editors. Disease Control Priorities in Developing Countries (2nd Edition). New York: Oxford University Press; 2006. p. 887-906.

2. Medina-Mora ME, Monteiro M, Room R, Rehm J, Jernigan D, Sánchez-Moreno D, et al. Alcohol Use and Alcohol Use Disorders In: Patel V, Chisholm D, Dua T, Laxminarayan R, Medina-Mora ME, editors. Disease Control Priorities, 3rd Edition Volume Disease Control Priorities for mental, neurological and substance use disorders. Washington, D.C.: World Bank; Forthcoming.

3. van der Feltz-Cornelis CM, Sarchiapone M, Postuvan V, Volker D, Roskar S, Grum AT, et al. Best practice elements of multilevel suicide prevention strategies: a review of systematic reviews. Crisis. 2011;32(6):319-33. PubMed PMID: 21945840. Pubmed Central PMCID: 3306243.

4. Vijayakumar L, Phillips M, Silverman M, Gunnell D, Carli V. Suicide In Low And Middle Income Countries In: Patel V, Chisholm D, Dua T, Laxminarayan R, Medina-Mora ME, editors. Disease Control Priorities, 3rd Edition Volume Disease Control Priorities for mental, neurological and substance use disorders. Washington, D.C.: World Bank; Forthcoming.

5. Save the Children UK. A rough guide to child protection systems. United Kingdom: 2009.

6. Fluke JD, Goldman PS, Shriberg J, Hillis SD, Yun K, Allison S, et al. Systems, strategies, and interventions for sustainable long-term care and protection of children with a history of living outside of family care. Child abuse & neglect. 2012 Oct;36(10):722-31. PubMed PMID: 23102720.

7. Medina MT, Aguilar‐Estrada RL, Alvarez A, Durón RM, Martínez L, Dubón S, et al. Reduction in rate of epilepsy from neurocysticercosis by community interventions: the Salama, Honduras study. Epilepsia. 2011;52(6):1177-85.

8. Clement S, Lassman F, Barley E, Evans-Lacko S, Williams P, Yamaguchi S, et al. Mass media interventions for reducing mental health-related stigma. The Cochrane database of systematic reviews. 2013;7:CD009453. PubMed PMID: 23881731. Epub 2013/07/25. eng.

9. Corrigan PW, Morris SB, Michaels PJ, Rafacz JD, Rusch N. Challenging the public stigma of mental illness: a meta-analysis of outcome studies. Psychiatric services. 2012 Oct;63(10):963-73. PubMed PMID: 23032675.

10. Dunion L, Gordon L. Tackling the attitude problem. The achievements to date of Scotland's' see me'anti-stigma campaign. Mental health today (Brighton, England). 2005:22.

11. Evans-Lacko S, Malcolm E, West K, Rose D, London J, Rusch N, et al. Influence of Time to Change's social marketing interventions on stigma in England 2009-2011. BrJPsychiatry Suppl. 2013;55:s77-s88.

12. Thornicroft C, Wyllie A, Thornicroft G, Mehta N. Impact of the "Like Minds, Like Mine" anti-stigma and discrimination campaign in New Zealand on anticipated and experienced discrimination. Aust N Z J Psychiatry. 2014 Apr;48(4):360-70. PubMed PMID: 24253359. Epub 2013/11/21. eng.

13. Finkelstein J, Lapshin O, Wasserman E. Randomized study of different anti-stigma media. Patient Educ Couns. 2008 May;71(2):204-14. PubMed PMID: 18289823. Epub 2008/02/22. eng.

14. Nytro K, Saksvik PO, Mikkelsen A, Bohle P, Quinlan M. An appraisal of key factors in the implemention for occuaptional stress interventions. . Work and Stress. 2000;14(3):213-25.

15. Probst TM, Gold D, Caborn J. A preliminary evaluation of SOLVE: addressing psychosocial problems at work. Journal of occupational health psychology. 2008 Jan;13(1):32-42. PubMed PMID: 18211167.

16. Godard C, Chevalier A, Lecrubier Y, Lahon G. APPRAND programme: An intervention to prevent relapses of anxiety and depressive disorders – First results of a medical health promotion intervention in a population of employees. European Psychiatry. 2006;21(7):451-9.

17. Kitchener B, Jorm A. Mental health first aid training in a workplace setting: A randomized controlled trial. BMC psychiatry. 2004;4:23.

18. Page MJ, Paramore LC, Doshi D, Rupnow MF. Evaluation of resource utilization and cost burden before and after an employer-based migraine education program. Journal of Occupational and Environmental Medicine. 2009;51(2):213-20.

19. Schneider WJ, Furth PA, Blalock TH, Sherrill TA. A pilot study of a headache program in the workplace: the effect of education. Journal of occupational and environmental medicine. 1999;41(3):202-9.

20. Crowther R, Marshall M, Bond G, Huxley P. Helping people with severe mental illness to obtain work: Systematic review. British Medical Journal. 2001;322:204-8.

21. Dickson K, Gough D. Supporting people in accessing meaningful work: Recovery approaches in community-based adult mental health services. London: Social Care Institute for Evidence, 2008.

22. McDaid D. Mental Health in Workplace Settings. Consensus paper. Luxembourg: European Communities, 2008.

23. British Occupational Health Research Foundation (BOHRF). Workplace interventions for people with common mental health problems: Evidence review and recommendations. London: BOHRF, 2005.

24. Hill D, Lucy D, Tyers C, James L. What works at work. Review of Evidence Assessing the Effectiveness of Workplace Interventions to Prevent and Manage Common Healthcare Problems. 2007.

25. Seymour L. Common mental health problems at work: What we now know about successful interventions. A progress review. London: Sainsburys Centre for Mental Health, 2010.

26. Pinfold V, Toulmin H, Thornicroft G, Huxley P, Farmer P, Graham T. Reducing psychiatric stigma and discrimination: evaluation of educational interventions in UK secondary schools. The British Journal of Psychiatry. 2003;182(4):342-6.

27. Swartz KL, Kastelic EA, Hess SG, Cox TS, Gonzales LC, Mink SP, et al. The effectiveness of a school-based adolescent depression education program. Health Education & Behavior. 2010;37(1):11-22.

28. Watson AC, Otey E, Westbrook AL, Gardner AL, Lamb TA, Corrigan PW, et al. Changing middle schoolers' attitudes about mental illness through education. Schizophrenia Bulletin. 2004;30(3):563-72.

29. Rahman A, Mubbashar MH, Gater R, Goldberg D. Randomised trial of impact of school mental-health programme in rural Rawalpindi, Pakistan. Lancet. 1998 Sep 26;352(9133):1022-5. PubMed PMID: 9759745.

30. Durlak JA, Weissberg RP, Dymnicki AB, Taylor RD, Schellinger KB. The impact of enhancing students' social and emotional learning: a meta-analysis of school-based universal interventions. Child development. 2011 Jan-Feb;82(1):405-32. PubMed PMID: 21291449.

31. Lister-Sharp D, Chapman S, Stewart-Brown S, Sowden A. Health promoting schools and health promotion in schools: two systematic reviews. Health technology assessment. 1999;3(22):1-207. PubMed PMID: 10683593.

32. NICE. Social and emotional wellbeing in secondary education. NICE public health guidance 20. United Kingdom: National Institute of Health and Clinical Excellence. , 2009.

33. Tennant R, Goens C, Barlow J, Day C, Stewart-Brown S. A systematic review of reviews of interventions to promote mental health and prevent mental health problems in children and young people. Journal of Public Mental Health 2007;6(1):25-32.

34. Weare K, Nind M. Mental health promotion and problem prevention in schools: what does the evidence say? Health promotion international. 2011 Dec;26 Suppl 1:i29-69. PubMed PMID: 22079935.

35. Wells J, Barlow J, Stewart-Brown S. A systematic review of universal approaches to mental health promotion in schools. Health Education. 2003;103(4):197-220.

36. Barry MM, Clarke AM, Jenkins R, Patel V. A systematic review of the effectiveness of mental health promotion interventions for young people in low and middle income countries. BMC public health. 2013;13(1):835.

37. De Villiers M, Van den Berg H. The implementation and evaluation of a resiliency programme for children. South African Journal of Psychology. 2012;42(1):93-102.

38. Smith EA, Palen L-A, Caldwell LL, Flisher AJ, Graham JW, Mathews C, et al. Substance use and sexual risk prevention in Cape Town, South Africa: an evaluation of the HealthWise program. Prevention Science. 2008;9(4):311-21.

39. Srikala B, Kishore KK. Empowering adolescents with life skills education in schools–School mental health program: Does it work? Indian journal of psychiatry. 2010;52(4):344.

40. Clarke GN, Hawkins W, Murphy M, Sheeber LB, Lewinsohn PM, Seeley JR. Targeted prevention of unipolar depressive disorder in an at-risk sample of high school adolescents: A randomized trial of a group cognitive intervention. Journal of the American Academy of Child & Adolescent Psychiatry. 1995;34(3):312-21.

41. Jaycox LH, Reivich KJ, Gillham J, Seligman ME. Prevention of depressive symptoms in school children. Behaviour research and therapy. 1994;32(8):801-16.

42. Shucksmith J, Summerbell C, Jones S, Whittaker V. Mental wellbeing of children in primary education (targeted/indicated activities). Teeside: University of Teeside. 2007.

43. Fazel M, Patel V, Thomas S, Tol W. Mental health interventions in schools in low-income and middle-income countries. The Lancet Psychiatry. 2014;1(5):388-98.

44. Tol WA, Komproe IH, Jordans MJ, Ndayisaba A, Ntamutumba P, Sipsma H, et al. School-based mental health intervention for children in war-affected Burundi: a cluster randomized trial. BMC medicine. 2014;12(1):56.

45. Jorm AF, Kitchener BA, Sawyer MG, Scales H, Cvetkovski S. Mental health first aid training for high school teachers: a cluster randomized trial. BMC psychiatry. 2010;10:51. PubMed PMID: 20576158. Pubmed Central PMCID: 2908569.

46. Becker AE, Thomas JJ, Bainivualiku A, Richards L, Navara K, Roberts AL, et al. Adaptation and evaluation of the Clinical Impairment Assessment to assess disordered eating related distress in an adolescent female ethnic Fijian population. The International journal of eating disorders. 2010 Mar;43(2):179-86. PubMed PMID: 19308992. Pubmed Central PMCID: 2896728.

47. Goel S, Singh N, Lal V, Singh A. Evaluating the impact of comprehensive epilepsy education programme for school teachers in Chandigarh city, India. Seizure: the journal of the British Epilepsy Association. 2014;23(1):41-6.

48. Vieira MA, Gadelha AA, Moriyama TS, Bressan RA, Bordin IA. Evaluating the effectiveness of a training program that builds teachers' capability to identify and appropriately refer middle and high school students with mental health problems in Brazil: an exploratory study. BMC public health. 2014;14(1):210. PubMed PMID: 24580750. Pubmed Central PMCID: 3975921.

49. DuPaul GE, TL.; Vilardo, B. The Effects of School-Based Interventions for Attention Deficit Hyperactivity Disorder: A Meta-Analysis 1996-2010. School Psychology Review. 2012;41:387-412.

50. Kutcher S, Wei YF. Mental health and the school environment: secondary schools, promotion and pathways to care. Curr Opin Psychiatr. 2012 Jul;25(4):311-6. PubMed PMID: WOS:000305919200009. English.

51. Mychailyszyn MP BD, Read KL, Kendall PC. Cognitive-Behavioral School-Based Interventions for Anxious and Depressed Youth: A Meta-Analysis of Outcomes. Clinical Psychology Science and Practice. 2012;52(11):1124-33.

52. Araya R, Fritsch R, Spears M, Rojas G, Martinez V, Barroilhet S, et al. School Intervention to Improve Mental Health of Students in Santiago, Chile A Randomized Clinical Trial. Jama Pediatr. 2013 Nov;167(11):1004-10. PubMed PMID: WOS:000329842300011. English.

53. Jordans MJ, Komproe IH, Tol WA, Kohrt BA, Luitel NP, Macy RD, et al. Evaluation of a classroom‐based psychosocial intervention in conflict‐affected Nepal: a cluster randomized controlled trial. Journal of Child Psychology and Psychiatry. 2010;51(7):818-26.

54. Tol WA, Komproe IH, Susanty D, Jordans MJ, Macy RD, De Jong JT. School-based mental health intervention for children affected by political violence in Indonesia: a cluster randomized trial. JAMA : the journal of the American Medical Association. 2008;300(6):655-62.

55. Tol WA, Komproe IH, Jordans MJ, Vallipuram A, Sipsma H, Sivayokan S, et al. Outcomes and moderators of a preventive school‐based mental health intervention for children affected by war in Sri Lanka: a cluster randomized trial. World psychiatry : official journal of the World Psychiatric Association. 2012;11(2):114-22.

56. Barlow J, Parsons J, Stewart-Brown S. Preventing emotional and behavioural problems: the effectiveness of parenting programmes with children less than 3 years of age. Child: care, health and development. 2005 Jan;31(1):33-42. PubMed PMID: 15658964.

57. Cooper PJ, Tomlinson M, Swartz L, Landman M, Molteno C, Stein A, et al. Improving quality of mother-infant relationship and infant attachment in socioeconomically deprived community in South Africa: randomised controlled trial. BMJ: British Medical Journal. 2009;338.

58. Jin X, Sun Y, Jiang F, Ma J, Morgan C, Shen X. "Care for Development" intervention in rural China: a prospective follow-up study. Journal of developmental and behavioral pediatrics : JDBP. 2007 Jun;28(3):213-8. PubMed PMID: 17565288.

59. Mejia A, Calam R, Sanders MR. A review of parenting programs in developing countries: opportunities and challenges for preventing emotional and behavioral difficulties in children. Clinical child and family psychology review. 2012 Jun;15(2):163-75. PubMed PMID: 22427004.

60. Rahman A, Iqbal Z, Roberts C, Husain N. Cluster randomized trial of a parent-based intervention to support early development of children in a low-income country. Child: care, health and development. 2009 Jan;35(1):56-62. PubMed PMID: 18991970.

61. Wendland-Carro J, Piccinini CA, Millar WS. The role of an early intervention on enhancing the quality of mother-infant interaction. Child development. 1999 May-Jun;70(3):713-21. PubMed PMID: 10368917.

62. Walker S, Susan M. Chang. Effectiveness of parent support programmes in enhancing learning in the under-3 age group. Early Childhood Matters. 2013;120:45 - 9.

63. Kaminski JW, Valle LA, Filene JH, Boyle CL. A meta-analytic review of components associated with parent training program effectiveness. Journal of abnormal child psychology. 2008 May;36(4):567-89. PubMed PMID: 18205039. Epub 2008/01/22. eng.

64. Oveisi S, Ardabili HE, Dadds MR, Majdzadeh R, Mohammadkhani P, Rad JA, et al. Primary prevention of parent-child conflict and abuse in Iranian mothers: a randomized-controlled trial. Child abuse & neglect. 2010 Mar;34(3):206-13. PubMed PMID: 20207004.

65. Vasquez M, Meza, L. Almandarez, O., Santos, A., Matute, R.C., Canaca, L.D., Cruz, A., Acosta, S., Garcia Bacilla, M.A., Wilson, L., Azuero, A., Tami, I., Holcomb, L., Saenz, K. . Evaluation of a strengthening families (Familias Fuertes) intervention for parents and adolescents in Honduras. Southern Online Journal of Nursing Research. 2010;10(3).

66. Fayyad JA, Farah L, Cassir Y, Salamoun MM, Karam EG. Dissemination of an evidence-based intervention to parents of children with behavioral problems in a developing country. European child & adolescent psychiatry. 2010 Aug;19(8):629-36. PubMed PMID: 20169380.

67. Jewkes R, Nduna M, Levin J, Jama N, Dunkle K, Puren A, et al. Impact of stepping stones on incidence of HIV and HSV-2 and sexual behaviour in rural South Africa: cluster randomised controlled trial. BMJ: British Medical Journal. 2008;337.

68. Kim J, Ferrari G, Abramsky T, Watts C, Hargreaves J, Morison L, et al. Assessing the incremental effects of combining economic and health interventions: the IMAGE study in South Africa. Bulletin of the World Health Organization. 2009;87(11):824-32.

69. Pronyk PM, Hargreaves JR, Kim JC, Morison LA, Phetla G, Watts C, et al. Effect of a structural intervention for the prevention of intimate-partner violence and HIV in rural South Africa: a cluster randomised trial. The lancet. 2006;368(9551):1973-83.

70. Ssewamala FM, Han CK, Neilands TB. Asset ownership and health and mental health functioning among AIDS-orphaned adolescents: findings from a randomized clinical trial in rural Uganda. Soc Sci Med. 2009 Jul;69(2):191-8. PubMed PMID: 19520472. Pubmed Central PMCID: 2819297.

71. Brady M, Assaad R, Ibrahim B, Salem A, Salem R. Providing new opportunities to adolescent girls in socially conservative settings: the Ishraq program in rural Upper Egypt. 2007.

72. Anderson LM, Shinn C, Fullilove MT, Scrimshaw SC, Fielding JE, Normand J, et al. The effectiveness of early childhood development programs. A systematic review. American journal of preventive medicine. 2003 Apr;24(3 Suppl):32-46. PubMed PMID: 12668197.

73. Nelson G, Westhues A, MacLeod J. A meta-analysis of longitudinal research on preschool prevention programs for children. 6. 2003;31.

74. Schweinhart LJ, Montie J, Xiang Z, Barnett WS, Belfield CR, Nores M. Lifetime effects: the High/Scope Perry Preschool study through age 40. 2005.

75. Aboud FE. Evaluation of an early childhood preschool program in rural Bangladesh. Early Childhood Research Quarterly. 2006;21:46-60.

76. Cueto S, Guerrero G, Leon J, Zevallos A, Sugimaru C. Promoting early childhood development through a public programme: Wawa Wasi in Peru. Oxford: Oxford, UK Young Lives, Department of International Development, 2009.

77. Kagitcibasi C, Sunar D, Bekman S. Long-term effects of early intervention: Turkish low-income mothers and children. Journal of Applied Developmental Psychology. 2001;22(4):333-61.

78. Kagitcibasi C, Sunar D, Bekman S, Baydar N, Cemalcilar Z. Continuing effects of early enrichment in adult life: The Turkish Early Enrichment Project 22 years later. Journal of Applied Developmental Psychology. 2009;30(6):764-79.

79. Han HR, Park SY, Song H, Kim M, Kim KB, Lee HB. Feasibility and Validity of Dementia Assessment by Trained Community Health Workers Based on Clinical Dementia Rating. Journal of the American Geriatrics Society. 2013;61(7):1141-5.

80. Hansson L, Markstrom U. The effectiveness of an anti-stigma intervention in a basic police officer training programme: a controlled study. BMC psychiatry. 2014;14:55. PubMed PMID: 24568685. Pubmed Central PMCID: 3937239.

81. Jorm AF, Kitchener BA, O'Kearney R, Dear KB. Mental health first aid training of the public in a rural area: a cluster randomized trial [ISRCTN53887541]. BMC psychiatry. 2004;4:33. PubMed PMID: 15500695. Pubmed Central PMCID: 526774.

82. Krameddine Y DD, Robert H, Silverstone P. A novel training program for police officers that improves interactions with mentally ill individuals and is cost-effective. Frontiers in Psychiatry. 2013.

83. Teller JL, Munetz MR, Gil KM, Ritter C. Crisis intervention team training for police officers responding to mental disturbance calls. Psychiatric services. 2006 Feb;57(2):232-7. PubMed PMID: 16452701.

84. Watson AC, Morabito MS, Draine J, Ottati V. Improving police response to persons with mental illness: a multi-level conceptualization of CIT. International journal of law and psychiatry. 2008 Aug-Sep;31(4):359-68. PubMed PMID: 18632154. Pubmed Central PMCID: 2655327.

85. Chibanda D, Mesu P, Kajawu L, Cowan F, Araya R, Abas MA. Problem-solving therapy for depression and common mental disorders in Zimbabwe: piloting a task-shifting primary mental health care intervention in a population with a high prevalence of people living with HIV. BMC public health. 2011;11:828. PubMed PMID: 22029430. Pubmed Central PMCID: 3210104.

86. Lehman AF, Dixon LB, Kernan E, DeForge BR, Postrado LT. A randomized trial of assertive community treatment for homeless persons with severe mental illness. Archives of general psychiatry. 1997 Nov;54(11):1038-43. PubMed PMID: 9366661.

87. Marshall M, Lockwood A. Assertive community treatment for people with severe mental disorders. The Cochrane database of systematic reviews. 2000 (2):CD001089. PubMed PMID: 10796415.

88. Salyers MP, McGuire AB, Rollins AL, Bond GR, Mueser KT, Macy VR. Integrating assertive community treatment and illness management and recovery for consumers with severe mental illness. Community mental health journal. 2010 Aug;46(4):319-29. PubMed PMID: 20077006.

89. Chatterjee S, Naik S, John S, Dabholkar H, Balaji M, Koschorke M, et al. Effectiveness of a community-based intervention for people with schizophrenia and their caregivers in India (COPSI): a randomised controlled trial. Lancet. 2014 Apr 19;383(9926):1385-94. PubMed PMID: 24612754.

90. Koolaee AK, Etemadi A. The outcome of family interventions for the mothers of schizophrenia patients in Iran. The International journal of social psychiatry. 2010 Nov;56(6):634-46. PubMed PMID: 19734180.

91. Kulhara P, Chakrabarti S, Avasthi A, Sharma A, Sharma S. Psychoeducational intervention for caregivers of Indian patients with schizophrenia: a randomised-controlled trial. Acta psychiatrica Scandinavica. 2009 Jun;119(6):472-83. PubMed PMID: 19032700.

92. Li Z, Arthur D. Family education for people with schizophrenia in Beijing, China: randomised controlled trial. The British journal of psychiatry : the journal of mental science. 2005 Oct;187:339-45. PubMed PMID: 16199793.

93. Ran MS, Xiang MZ, Chan CL, Leff J, Simpson P, Huang MS, et al. Effectiveness of psychoeducational intervention for rural Chinese families experiencing schizophrenia--a randomised controlled trial. Social psychiatry and psychiatric epidemiology. 2003 Feb;38(2):69-75. PubMed PMID: 12563548.

94. Sharif F, Shaygan M, Mani A. Effect of a psycho-educational intervention for family members on caregiver burdens and psychiatric symptoms in patients with schizophrenia in Shiraz, Iran. BMC psychiatry. 2012;12:48. PubMed PMID: 22632135. Pubmed Central PMCID: 3441201.

95. Farooq S, Nazar Z, Irfan M, Akhter J, Gul E, Irfan U, et al. Schizophrenia medication adherence in a resource-poor setting: randomised controlled trial of supervised treatment in out-patients for schizophrenia (STOPS). The British journal of psychiatry : the journal of mental science. 2011 Dec;199(6):467-72. PubMed PMID: 22130748.

96. Maneesakorn S, Robson D, Gournay K, Gray R. An RCT of adherence therapy for people with schizophrenia in Chiang Mai, Thailand. Journal of clinical nursing. 2007 Jul;16(7):1302-12. PubMed PMID: 17584349.

97. Rahman A, Malik A, Sikander S, Roberts C, Creed F. Cognitive behaviour therapy-based intervention by community health workers for mothers with depression and their infants in rural Pakistan: a cluster-randomised controlled trial. Lancet. 2008 Sep 13;372(9642):902-9. PubMed PMID: 18790313. Pubmed Central PMCID: 2603063.
